# The extensive erythrocyte-plasma partitioning of trametinib – implications for pharmacokinetic studies and therapeutic drug monitoring

**DOI:** 10.1007/s43440-026-00840-y

**Published:** 2026-02-19

**Authors:** Bence János Chriszt, Zoltán Köllő, Orsolya Geda, Éva Csöndör, Róbert Farkas, Barna Vásárhelyi, Miklós Garami, Gellért Balázs Karvaly

**Affiliations:** 1 Department of Laboratory Medicine, Semmelweis University, Floor 14, 4 Nagyvárad tér, Budapest, H-1089 Hungary; 2 Tűzoltó Street Department, Pediatric Center, Semmelweis University, Budapest, Hungary

**Keywords:** Therapeutic drug monitoring, Pharmacokinetics, Oncology, Anticancer drugs

## Abstract

**Background:**

Trametinib (TRT) is a kinase inhibitor displaying extensive erythrocyte-plasma partitioning (EPP). Plasma concentrations have been evaluated in most pharmacokinetic studies and for therapeutic drug monitoring (TDM), with controversial outcomes. We investigated EPP to evaluate its impact on TRT pharmacokinetics and laboratory assays.

**Methods:**

TRT concentrations were monitored in spiked whole blood, in plasma and erythrocytes separated from spiked whole blood, and in plasma spiked directly. The erythrocyte-plasma partitioning coefficient (K_e/p_) was established at 38 °C, room temperature (RT), and 2–8 °C. TRT concentrations and K_e/p_ were assessed in samples collected from a pediatric patient receiving TRT and in the steady state. Assays were performed using liquid chromatography-mass spectrometry and an in-house method validated for plasma TRT assays.

**Results:**

TRT was chemically stable in whole blood and plasma at RT and 2–8 °C. The TRT content of erythrocytes varied by concentration and storage temperature. At 20 ng/mL, K_e/p_ increased remarkably at RT and 38 °C, but remained unchanged at 2–8 °C. At 60 ng/mL, K_e/p_ increased throughout the 4-hour time frame, though to a considerably lesser extent. In in vivo samples, K_e/p_ showed a strong multivariate correlation with time after dose intake and the product of dose and hematocrit at 7.33–15.7 h postdose (*r* = 0.987). TRT also exerted temperature-dependent diffusion from erythrocytes into TRT-free plasma.

**Conclusions:**

EPP of TRT is related to concentration, temperature, and time in the therapeutic concentration range. Parallel whole blood and plasma TRT assays may therefore be useful for clinical pharmacokinetic studies and TDM.

**Supplementary Information:**

The online version contains supplementary material available at 10.1007/s43440-026-00840-y.

## Introduction

Trametinib (TRT) is a small-molecule selective mitogen-activated protein kinase inhibitor employed for the targeted therapy of various malignancies, including melanoma and non-small cell lung cancer, that result from mutations (such as V600E and V600K single-nucleotide polymorphisms or KIAA1549::BRAF fusion) in the B-Raf proto-oncogene [1]. By supporting individualized dosing regimens, therapeutic drug monitoring (TDM) of TRT can offer benefits, given the limited clinical experience with this substance, the high costs associated with its administration, and the risk of patient nonadherence, inadequate dosing, or the development of severe adverse effects [2, 3]. 

The extensive erythrocyte-plasma partitioning (EPP) of TRT was described shortly after its first clinical approval [4, 5]. This finding has nevertheless received little attention, and plasma TRT concentrations have been measured in almost all clinical pharmacokinetic and TDM studies. For such studies, the analysis of drugs with a blood-to-plasma concentration ratio exceeding one has been recommended using whole blood, and this recommendation has been implemented for various substances, including immunosuppressants (such as tacrolimus or cyclosporine A), hydroxychloroquine and its metabolites, as well as vitamins B1 and B6 [6–10]. Discrepancies have been revealed during attempts to estimate plasma TRT concentrations from assays of dried, volumetric absorptive whole-blood microsamples, including the identification of a moderate correlation (*r* = 0.581) between observed and predicted plasma TRT concentrations, in sharp contrast to that found for other protein kinase inhibitors such as cabozantinib, dabrafenib, nilotinib, and ruxolitinib (*r* = 0.943–0.947) [11–13]. Such a poor correlation indicates that neither erythrocyte saturation nor a constant equilibrium between erythrocytes and plasma was reached for TRT. Monitoring plasma TRT concentrations has repeatedly demonstrated significant intra-individual variability in TRT pharmacokinetics, and, ultimately, the clinical value of plasma TRT monitoring based on the analysis of trough samples has been debated [11, 14–16].

The EPP of other kinase inhibitors has been investigated. At therapeutic concentrations, a small fraction of imatinib enters erythrocytes, but coadministration with everolimus dramatically increases its extent of penetration. The relationship between total blood imatinib concentration and the partition ratio has proven inconsistent in such cases, showing significant inter-individual variability and necessitating the parallel monitoring of whole blood and plasma concentrations to assess its availability [17]. In vitro, dasatinib was taken up by erythrocytes, which subsequently triggered the apoptosis of target leukocytes [18]. The extensive EPP of alectinib and sunitinib was confirmed by comparing plasma and dried whole-blood microsample concentrations [19]. The partitioning characteristics of additional kinase inhibitors that enter erythrocytes to a lesser extent have been described in information documents published by regulatory authorities [20–22].

In a preliminary experiment conducted to establish the bench-top stability of TRT in spiked whole blood at room temperature (RT) and 2–8 °C, we observed a slow but steady decrease of TRT concentrations in plasma. Since no degradation occurred in plasma spiked directly, we hypothesized that a slow transfer of TRT into erythrocytes had taken place. In the current study, we aimed to investigate the details of the EPP of TRT in spiked human blood, focusing on the impact of TRT concentration within the therapeutic range, as well as sample temperature. We also aimed to extend this investigation to steady-state EPP by evaluating TRT concentrations in whole blood-plasma sample pairs obtained from a patient on chronic TRT therapy.

## Materials and methods

### Ethical background

This work was part of a monocentric, non-randomized, open-label cohort study (HUPON-2023-002) conducted at the Pediatric Center, Semmelweis University (Budapest, Hungary) and approved by the National Center for Public Health and Pharmacy of Hungary (identifier BM/13218-1/2023). A letter of approval was issued separately by the Regional and Institutional Committee of Science and Research Ethics, Semmelweis University (Budapest, Hungary) regarding self-sampling to five authors of this work (ref. SE RKEB 175/2023). All participants, or their legal guardians, had provided their written informed consent.

Blood samples were donated voluntarily, without financial or any other interest except for deepening scientific knowledge, by five authors of the present work (B.C., E.Cs., Z.K., R.F., and G.B.K.) for the in vitro experiments based on the processing of spiked blood samples. No participants other than the volunteering authors were involved in these experiments. The volunteers did not ingest TRT, and did not provide any clinical information; the samples were used solely as blank whole blood for technical purposes. Each experiment, conducted on separate days, involved drawing a single tube of blood from the antecubital vein in a standard phlebotomy process.

A single pediatric patient receiving TRT was included for the evaluation of EPP in the steady state. Blood samples taken for plasma TDM, offered as a service by the Department of Laboratory Medicine, Semmelweis University (Budapest, Hungary), were used after 200 µL was separated for diagnostic testing. Legal guardians provided written informed consent for the publication of the clinical and demographic data, as well as the laboratory test results reported here. No patient information was collected beyond that typically recorded within the TDM service, and all patient samples were collected for diagnostic purposes. The outcomes of these measurements did not result in any therapeutic intervention.

### Chemicals and consumables

TRT (product number: C3822, 98% pure) and ^13^C_6_-TRT (C3820, 99% pure) were procured from Alsachim SAS (Illkirch, France). LC-MS grade solvents (acetonitrile, 02870-0-39-47; formic acid, 1219 L-0-79-61; methanol, 20740-0-39-47; and water, 3355 L-0-39-47) were purchased from Reanal Labor Kft. (Budapest, Hungary). Dimethyl sulfoxide (472301) was bought from Merck Kft. (Budapest, Hungary). Chromsystems^®^ Endocrine plasma control (0010) was obtained from ABL&E-Jasco Magyarország Kft. (Budapest, Hungary). Phenomenex Impact 96-well Protein Precipitation plates (CE0-7565), Phenomenex Strata 96-well deep-well collection plates with slot volumes of 2 mL (AH0-7194), and Phenomenex XB-C18 50 × 2.1 mm stationary phases containing particles with a mean diameter of 1.7 μm (00B-4498-AN) were provided by Gen-Lab Kft. (Budapest, Hungary).

### Sample collection for in vitro experiments

Blood was drawn at the clinical phlebotomy unit of the Department of Laboratory Medicine, Semmelweis University, by a certified phlebotomist in 3-mL Vacuette^®^ collection tubes containing edetate tripotassium (Greiner Bio-One Hungary Ltd., Mosonmagyaróvár, Hungary).

### Analytical methodology

The preparation of whole blood and plasma samples consisted of deproteinization with acetonitrile containing the internal standard ^13^C_6_-TRT (50 ng/mL), followed by a threefold dilution with water. Quantitation was performed using liquid chromatography-mass spectrometry. Six-point calibration was conducted using plasma spiked at 7-560 ng/mL. Analytical and experimental details are provided as Supplementary information 1 and 2, respectively. Method validation (methodology and results) is described in Supplementary information 3 and 4, with detailed validation results provided in Supplementary tables S1-S4.

### Loss of TRT from plasma after spiking whole blood or plasma directly

TRT was spiked at 25.2 ng/mL to whole blood immediately after phlebotomy, or to plasma previously separated by centrifugation (*n* = 5). Spiked samples were kept at RT or 2–8 °C for 6 h. Aliquots (150 µL) were transferred to microtubes at 0, 1, 2, 3, and 6 h. Whole blood was centrifuged at 1,100 x g (7 min) at RT, and plasma was separated for analysis.

### Penetration of TRT into erythrocytes at various concentrations and temperatures

To investigate the relationship between the concentration of TRT and its penetration into erythrocytes directly, 20.1, 60.3, or 101 ng/mL TRT was spiked to whole blood immediately after sample collection (*n* = 5). Samples were subsequently kept at RT or 2–8 °C for 2 h. Aliquots (150 µL) were centrifuged at 1,100 x g (7 min), and the supernatant was stored at − 70 °C until analysis. An equal volume of the remaining pellet was diluted threefold with water, and, after being kept at RT for one hour to achieve complete hemolysis, the mixture was centrifuged at 10,000 x *g* (10 min).

### Relative recovery of TRT in spiked whole blood, plasma separated from spiked whole blood, and plasma spiked directly

The relative recovery (RR) of TRT in whole blood spiked at 20.1, 60.3, or 101 ng/mL (*n* = 3), as well as in plasma separated from whole blood by centrifugation at 1,100 x g (7 min) or spiked directly, was compared in a 2-hour storage experiment conducted at RT. Aliquots were taken for analysis at 0, 0.5, 1, and 2 h.

### Relationship of EPP with TRT concentration and blood temperature

The relationship between sample temperature and EPP was investigated in a biphasic experiment. TRT was spiked to whole blood or plasma directly at target concentrations of 20 and 60 ng/mL, respectively. In the first phase, whole blood and plasma samples were shaken at 38 °C (300 rpm, condition A), or allowed to sit at RT (condition B) or 2–8 °C (condition C) for 2 h. In the second phase, samples previously kept at 38 °C were allowed to sit at RT (condition A◊condition B), those previously kept at RT were cooled to 2–8 °C (condition B◊condition C), and refrigerated samples were warmed to 38 °C (condition C◊condition A) for another 2 h. Plasma aliquots (65 µL) were separated from whole blood (150 µL) at 0, 0.5, 1.0, 2.0, 2.5, 3.0, and 4.0 h. Plasma-spiked samples (65 µL) underwent the same process to account for any chemical degradation of TRT. All plasma aliquots were stored at -70 °C until analysis.

### TRT assays in samples obtained in vivo after reaching steady state

TRT concentrations were determined in seven samples collected for TDM into a 3-mL collection tube containing edetate potassium from a male patient, aged 20.2 months at the start of the study, diagnosed with pilocytic astrocytoma (WHO grade 1, BRAF V600E mutation) at the Pediatric Center, Semmelweis University, who was receiving TRT as a 50 µg/mL oral solution once daily. The daily doses on the occasions of sample collection (therapy days 420, 428, 441, 458, 479, 510, and 521) were 350 µg, 400 µg, 450 µg, 450 µg, 350 µg, and, at the last two visits, 425 µg. The patient’s age and weight measured at subsequent visits were 20.2, 20.4, 20.9, 21.4, 22.1, 23.2, and 23.5 months, and 11.4, 11.4, 13.6, 12.6, 12.6, 12.2, and 12.5 kg, respectively. The comedications received at the time of inclusion were levetiracetam 400 mg twice daily, clobazam 2.5 mg/1.25 mg/2.5 mg per os, cholecalciferol 400 IU per os once daily, lactulose 3 g per os once daily, and iron hydroxide polymaltose 30 mg per os once daily. Hematocrit was established as part of routine diagnostics on a hematology analyzer, and was recorded at subsequent visits as 0.31, 0.28, 0.24, 0.26, 0.28, 0.24, and 0.34, respectively.

In vivo sampling was performed at 12.5, 9.58, 7.33, 9.00, 7.75, 8.17, and 15.7 h postdose, in this order. A single sample was collected on each occasion. Samples were transported for TRT assay within 1 h of collection and processed without delay upon receipt by the laboratory. One hundred µL whole blood was transferred to a microcentrifuge tube, while the centrifugation of a 150-µL aliquot took place at 1,100 x g (7 min), followed by the separation of 65 µL plasma. Single aliquots were assayed.

The sample collected on therapeutic day 479 was divided into 150-µL aliquots of whole blood and plasma, which were analyzed after storage at RT for 1, 2, 3, and 6 h. Aliquots were frozen at − 70 °C until analysis.

To verify if the back-diffusion of TRT from erythrocytes into plasma could take place, whole blood collected in vivo on therapy day 521 was centrifuged at 1,100 x g (7 min), and the supernatant was replaced with an equivalent volume of reconstituted TRT-free lyophilized human plasma (Chromsystems^®^ Endocrine Plasma control for Biogenic Amines in Plasma, ABL&E-Jasco Hungary Ltd., Budapest, Hungary). Samples were kept at 38 °C, RT, or 2–8 °C for 6 h. TRT concentrations were determined at 1, 2, 3, and 6 h.

### Data evaluation

RRs were calculated by comparing concentrations observed at each sampling time to those obtained in the 0-minute samples. Concentrations assayed in whole blood and blood cell pellets were multiplied by 1.42 to account for the solid material content (approximately 30%). This correction was necessary since calibration was performed using plasma calibrators. The erythrocyte-plasma partitioning coefficient (K_e/p_) was calculated as K_e/p_=[(1/Hct) x (c_s_/c_p_-1)] + 1 [23], where c_s_ and c_p_ are the concentrations spiked into whole blood, and those measured in the separated plasma, respectively, and Hct is the hematocrit.

### Statistical analysis

Means and standard deviations were calculated in experiments conducted with samples processed in parallel. Ordinary least squares linear regression was performed using Microsoft Excel 365 to explore the correlation between two continuous variables. Multivariate linear regression was performed using the lm() function of the *stats* package, while three-dimensional visualization was accomplished using the scatterplot3d() function of the *scatterplot3d* package of R [24].

## Results

### Loss of TRT from plasma after spiking whole blood or plasma directly

**Fig. 1:**
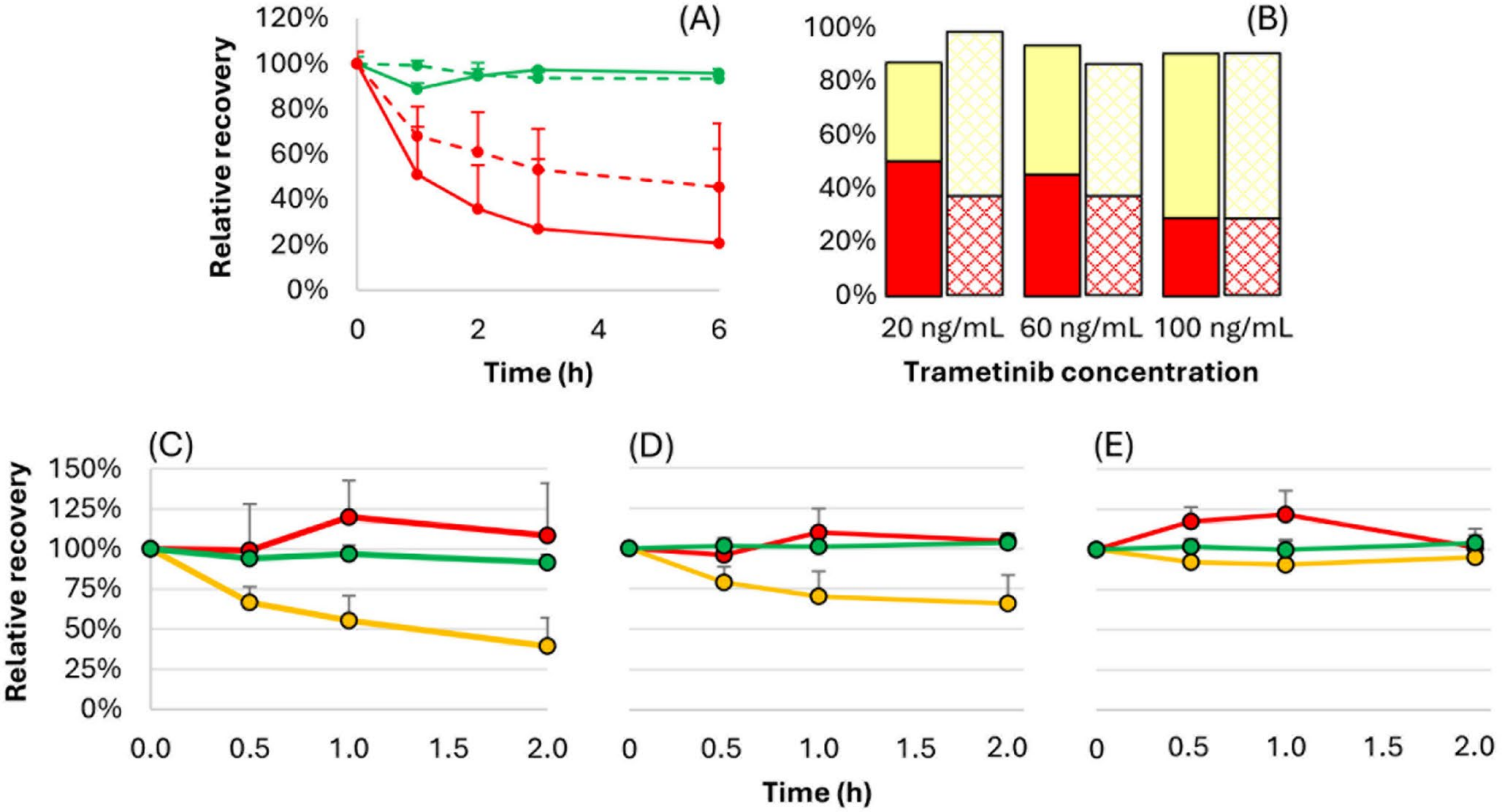
Mean relative recoveries (RR) of trametinib (TRT) in plasma, whole blood, and blood cell pellets, observed after spiking whole blood or plasma at 20, 60, or 100 ng/mL, and storage at room temperature (RT) or 2–8 °C. Assays were performed using liquid chromatography-tandem mass spectrometry and a method developed by the authors (see Supplementary information 1). RRs were calculated by dividing the measured concentrations by the initial concentrations and are expressed as percentages. The error bars show standard deviations. (**A**) Mean RRs in plasma (*n* = 5) during storage for 6 h at RT (solid lines) or 2–8 °C (dashed lines). Plasma was separated from spiked whole blood (red lines) or was spiked directly (green lines). (**B**) Mean RRs in plasma (yellow) and erythrocytes (red, *n* = 5). Samples were kept at RT (full bars) or 2–8 °C (bars with crosshatches). At 20 ng/mL, the mean RRs in the cellular pellet and in plasma were 50.4 ± 9.8% and 36.8%±7.8% at RT, as well as 36.6 ± 7.5% and 61.5 ± 5.0% at 2–8 °C, respectively. At 60 ng/mL, the RRs were 45.5 ± 3.4% and 48.2 ± 2.8% at RT, as well as 37.1 ± 3.1% and 49.4 ± 2.5% at 2–8 °C. At 100 ng/mL, the RRs were 29.2 ± 3.3% and 61.3 ± 5.6%, as well as 28.7 ± 2.0% and 61.9 ± 5.4%. Standard deviations are not shown in the plot. (**C**-**E**) Changes in mean RRs at RT over 2 h in spiked whole blood (red), plasma separated from spiked whole blood (yellow), and plasma spiked directly (green) at (**C**) 20 ng/mL, (**D**) 60 ng/mL, or (**E**) 100 ng/mL (*n* = 3)

The influence of time, storage temperature (RT or 2–8 °C), and concentration on the RRs after spiking whole blood or plasma, as well as the demonstration of the chemical stability of TRT in whole blood and plasma, are shown in Fig. 1. A.

### Penetration of TRT into erythrocytes at various concentrations and temperatures

The impact of concentration and storage temperature on TRT fractions in blood cells and plasma is displayed in Fig. 1. B.

### RR of TRT in whole blood, plasma separated from spiked whole blood, and plasma spiked directly

The RRs of TRT obtained in whole blood and plasma after spiking whole blood or plasma directly at 20, 60, or 100 ng/mL and storage for 2 h at RT are displayed in Fig. 1. C, D, and E, respectively.

### Relationship of EPP with TRT concentration and blood temperature

**Fig. 2:**
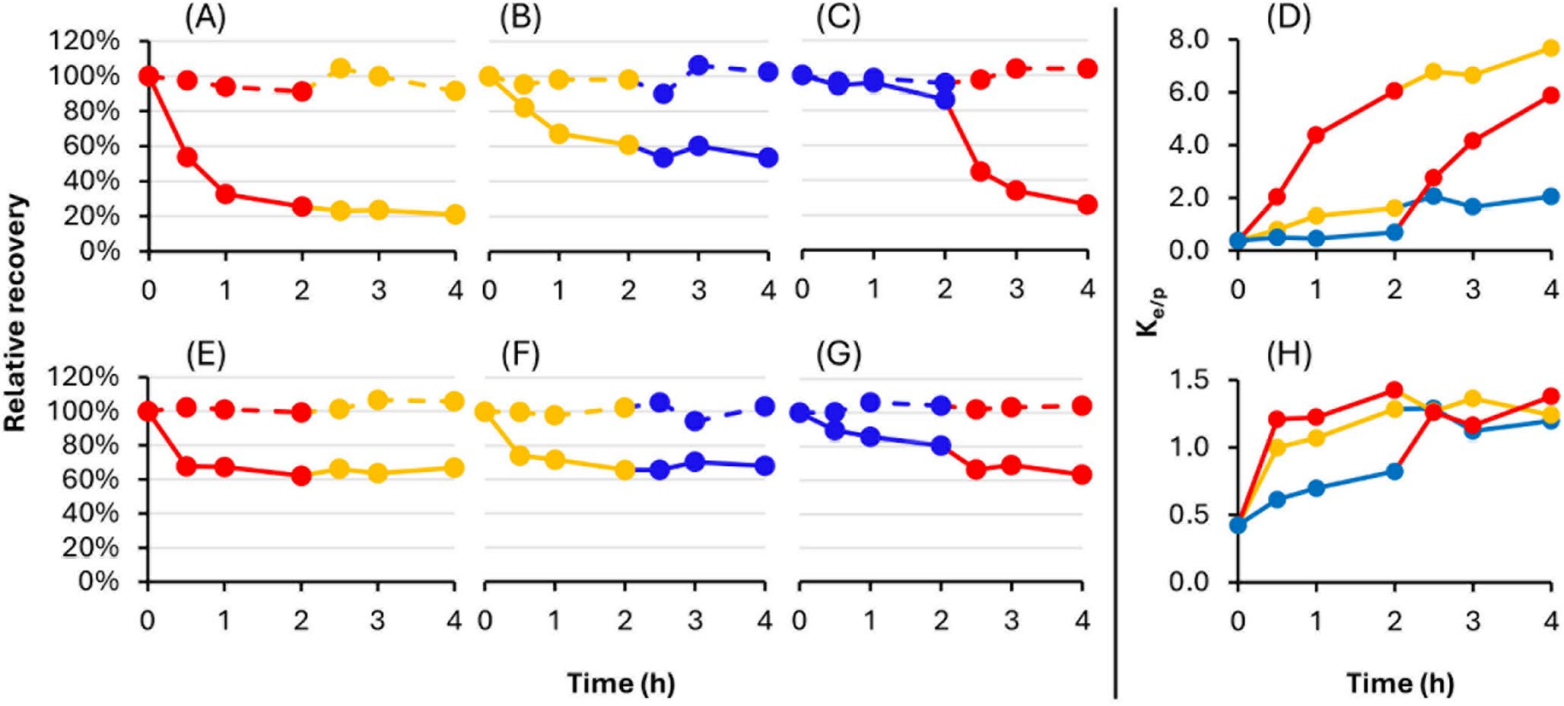
Trametinib (TRT) relative recoveries (RR) in plasma separated from spiked whole blood (solid lines) and in plasma spiked directly (dashed lines) during storage at 38 °C (red lines), room temperature (RT, orange lines), or 2–8 °C (blue lines) in a biphasic storage experiment. Whole blood concentrations were 26.4 ng/mL or 79.3 ng/mL; plasma concentrations were 20.3 ng/mL or 62.2 ng/L. Experiments were performed without replicates; the data points represent single measurements. Storage conditions were changed at 2 h from 38 °C to RT [(**A**) and (**E**)], from RT to 2–8 °C [(**B**) and (**F**)], or from 2–8 °C to 38 °C [(**C**) and (**G**)]. RRs were calculated by dividing the measured concentrations by the initial concentrations and are expressed as percentages. The erythrocyte-plasma partition coefficient (K_e/p_) was calculated using the equation K_e/p_=[(1/hematocrit) x (c_s_/c_p_-1)] + 1, where c_s_ was the concentration spiked into whole blood, and c_p_ was the concentration measured in the separated plasma [23]. The hematocrits of the low- and the high-level blood samples were 0.36 and 0.45, respectively. (**A**-**C**) RRs and (**D**) K_e/p_ values obtained at the low TRT levels. (**E**-**G**) RRs and (**H**) K_e/p_ values obtained at the high TRT levels

In the biphasic experiment, spiked whole-blood and plasma samples were sequentially incubated at various temperatures. RR and K_e/p_ showed different trends at 20 ng/mL and 60 ng/mL, and at various temperatures in plasma separated from spiked whole blood (Fig. 2). A K_e/p_ of 0.372 and 0.424, respectively, was observed immediately after spiking. In the condition A (38 °C)/condition B (RT) experiment, RRs in whole blood spiked at 20 ng/mL were 25.4% and 20.9% by the end of the first and second phases, respectively. K_e/p_ rose to 6.05 and then to 7.68. At 60 ng/mL, the RR was 62.2% at 2 h, with a K_e/p_ of 1.42, increasing to 66.8% at the end of the second phase, with the K_e/p_ decreasing to 1.24. In the condition B/condition C (2–8 °C) experiment, the RRs at 20 ng/mL were 61.0% (K_e/p_: 1.61) and 53.6% (K_e/p_: 2.05) at 2 and 4 h, respectively. At 60 ng/mL, RRs of 65.7% (K_e/p_: 1.28) and 68.0% (K_e/p_: 1.20) were recorded after two and four hours. In the condition C/condition A experiment, the RRs observed at the end of the first and second phases, respectively, were 85.9% (K_e/p_: 0.69) and 26.0% (K_e/p_: 5.89) at 20 ng/mL, as well as 80.5% (K_e/p_: 0.82) and 63.3% (K_e/p_: 1.38) at 60 ng/mL. In plasma spiked directly, the RRs were 90.0-106% and 94.1–107% at 20 and 60 ng/mL, respectively.

### TRT assays in samples obtained in vivo after reaching steady-state

**Fig. 3:**
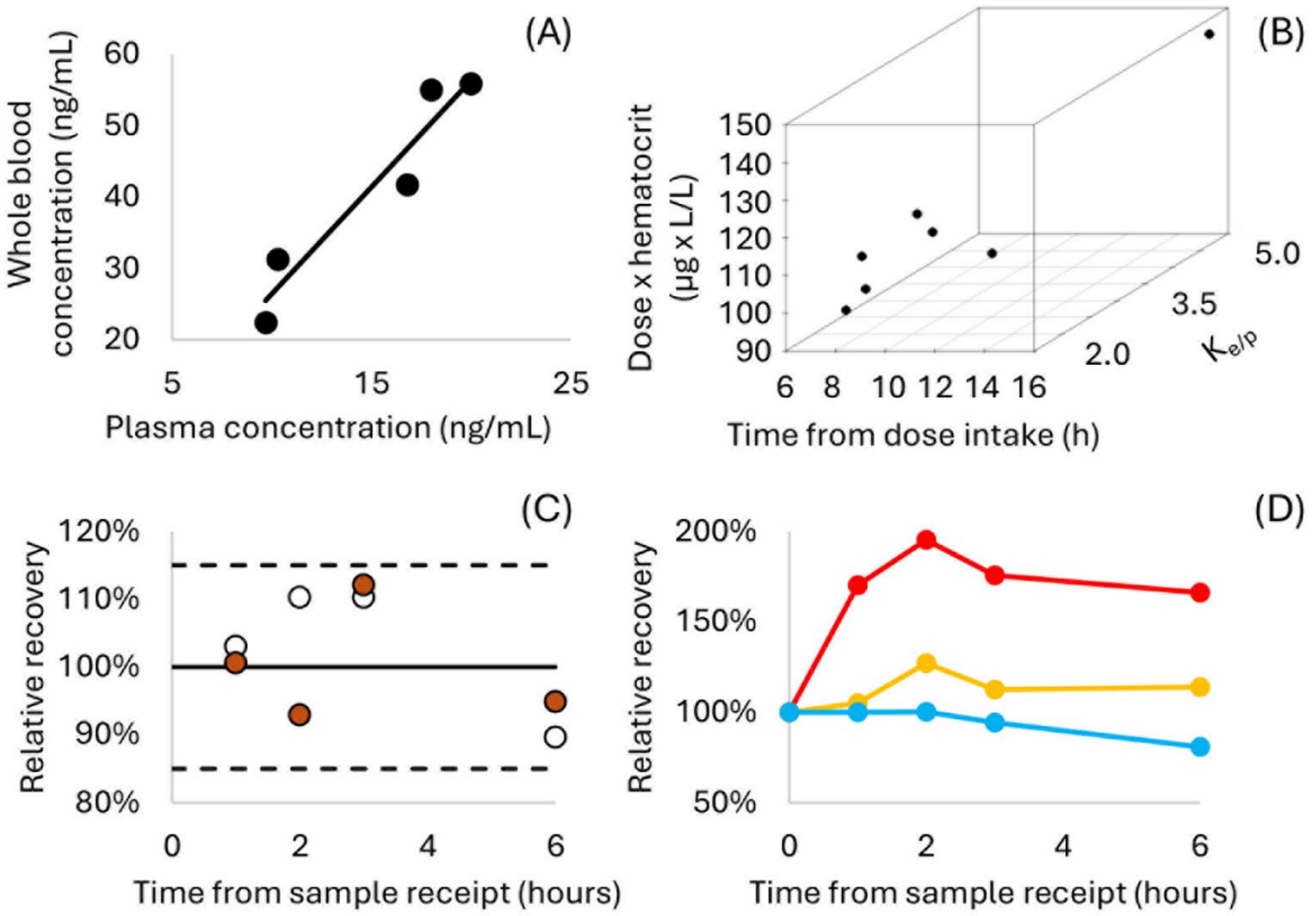
Results of the trametinib (TRT) assays performed in in vivo blood samples collected from a pediatric patient using a liquid chromatography-tandem mass spectrometry method developed by the authors (see Supplementary information 1). Relative recoveries (RR) were calculated by dividing the measured concentrations by those obtained at the beginning of sample storage and are expressed as percentages. (**A**) Correlation of whole blood and plasma TRT concentrations observed at 7.33–9.58 h postdose on days 428–521 of TRT therapy (the patient took oral doses of 350–450 µg, hematocrits: 0.24–0.28). The following regression equation was obtained: whole-blood concentration = plasma concentration х 2.994–3.533. Pearson’s correlation coefficient was 0.957. (**B**) Multivariate relationship of time, the product of dose and hematocrit, and the erythrocyte-plasma partitioning coefficient (K_e/p_) in samples collected at 7.33–15.7 h postdose on days 420–521 of TRT therapy. The TRT doses were 350–450 µg (hematocrits: 0.24–0.34). The coefficients of the regression equation were: intercept, -12.041; slope (K_e/p_ versus time), 0.231; slope (K_e/p_ versus dose x hematocrit), 0.202. Pearson’s correlation coefficient was 0.987. (**C**) RRs obtained during the storage of a whole blood sample (hematocrit: 0.28) drawn 7.75 h after the intake of a 350-µg oral dose of TRT on day 479 of TRT therapy at room temperature (RT) for six hours. RRs were calculated both in plasma (hollow circles) and whole blood (brown circles). (**D**) RRs of TRT in plasma over six hours of shaking three aliquots of a whole blood sample (hematocrit: 0.34) at 38 °C (red), RT (orange), or 2–8 °C (blue) after replacing patient plasma with an equivalent volume of TRT-free plasma. The patient had taken 425 µg TRT orally, and the sample was drawn at 15.7 h postdose

In samples obtained in vivo, plasma and whole blood TRT concentrations were essentially correlated in the five samples collected at 8.37 ± 0.82 h postdose (whole blood concentration = plasma concentration х 2.994–3.533; *r* = 0.957), with the mean concentration ratio (± standard deviation) being 2.74 ± 0.30 (the ratios obtained at 12.5 and 15.7 h were excluded from this calculation to minimize the impact of time on the relationship). In this time frame, the correlation between plasma and whole-blood concentrations and the dose was weak (*r* = 0.419 and 0.484, respectively). K_e/p_ displayed an essential multivariate correlation with time after dose intake and the product of dose and hematocrit (*r* = 0.976), but a weak correlation with the concentrations measured in plasma and whole blood (*r* = 0.317 and 0.387, respectively). In addition, K_e/p_ displayed a strong correlation with the whole blood-plasma concentration ratio (*r* = 0.968). TRT concentrations remained stable in plasma and whole blood at RT up to 6 h. After replacing plasma with blank plasma to investigate whether the EPP of TRT was bidirectional, TRT exerted temperature-dependent diffusion out of the erythrocytes at 25 and 38 °C, with concentrations increasing to 195% and 127% of the initial level, respectively (Fig. 3).

## Discussion

The results of the current study demonstrate that TRT undergoes a concentration-, temperature-, and time-dependent EPP in the therapeutic concentration range. Concurrently, equilibrium is reached rapidly at a supratherapeutic level (100 ng/mL). Since TRT partitioning can be bidirectional in this concentration range, changes in TRT concentration or blood temperature influence K_e/p_ and, consequently, plasma levels. In steady state, whole blood and plasma concentrations were strongly correlated, but the concentration ratios were not constant. Apparently, hematocrit should also be taken into account to establish a quantitative relationship in this respect, as demonstrated by the strong correlation between K_e/p_, time from dose intake, and the product of dose and hematocrit, along with a considerably weaker correlation between TRT concentrations and the dose (note that the range of doses was narrow).

The results also demonstrate that the concentration- and temperature-dependent partitioning of TRT may impact the results of laboratory assays and estimates of pharmacokinetic characteristics in plasma measurements. A clear distinction must be made between these two aspects of TRT partitioning. In vivo, substantial intra- and interindividual variability in the estimated pharmacokinetic characteristics of TRT can be expected. The variability may be substantial before the steady state is reached, during hematocrit changes, or after dose adjustment. This fact is clinically relevant, as TRT has a plasma half-life of approximately 4 days; therefore, attaining steady state or reaching a new steady state after dose adjustment may take 16–20 days. In vitro, cooling whole blood or separating plasma immediately after phlebotomy appears important to prevent unwanted changes in plasma concentrations, which may be especially pronounced before the steady state is reached. In comparison, the concurrent analysis of TRT in whole blood provides the important advantage of delivering more robust concentrations, eliminating in vitro preanalytical errors related to changes in EPP, and the opportunity to calculate K_e/p_.

The evaluation of spiked in vitro samples and samples obtained in vivo from a single patient poses a severe limitation to the interpretation of our results; nevertheless, our findings compare well with those observed earlier in in vitro and in vivo experiments using ^14^C-TRT [4, 5]. Another limitation is that the TRT dose range was very narrow and considerably lower than the standard daily dose (2 mg).

In view of the discouraging results from certain TDM studies focusing on plasma TRT concentrations, conducting TRT assays in whole blood and plasma in parallel, and including K_e/p_ and hematocrit in clinical pharmacokinetic models and calculations may be helpful for clinical decision-making.

## Supplementary Information

Below is the link to the electronic supplementary material.

Supplementary Material 1

## Data Availability

The data underlying this research are available from the authors upon reasonable request.

## Abbreviations

EPP: Erythrocyte-plasma partitioning
K_e/p_: Erythrocyte-plasma partitioning coefficient
RR: Relative recovery
RT: Room temperature
TDM: Therapeutic drug monitoring
TRT: Trametinib

## References

[1] Mekinist -summary of product characteristics. https://www.ema.europa.eu/en/documents/product-information/mekinist-epar-product-information_en.pdf.

[2] Ravix A, Bandiera C, Cardoso E, Lata-Pedreira A, Chtioui H, Decosterd LA, et al. Population pharmacokinetics of Trametinib and impact of nonadherence on drug exposure in oncology patients as part of the optimizing oral targeted anticancer therapies study. Cancers. 2024;16(12):2193. 10.3390/cancers16122193. 38927898 10.3390/cancers16122193 PMC11201946

[3] Van der Kleij MBA, Guchelaar NAD, Methijssen RHJ, Versluis J, Huitema ADR, Koolen SLW, Steeghs N. Therapeutic drug monitoring of kinase inhibitors in oncology. Clin Pharmacokinet. 2023;62(10):1333–64. 10.1007/s40262-023-01293-9. 37584840 10.1007/s40262-023-01293-9 PMC10519871

[4] Leonowens C, Pendry C, Bauman J, Young GC, Ho M, Henriquez F, et al. Concomitant oral and intravenous pharmacokinetics of trametinib, a MEK inhibitor, in subjects with solid tumors. Br J Clin Pharmacol. 2014;78(3):524–32. 10.1111/bcp.12373. 24606567 10.1111/bcp.12373 PMC4243903

[5] Ho MYK, Morris MJ, Pirhalla JL, Bauman JW, Pendry CB, Orford KW, et al. Trametinib, a first-in-class oral MEK inhibitor, mass balance study with limited enrollment of two male subjects with advanced cancers. Xenobiotica. 2014;44(4):352–68. 10.3109/00498254.2013.831143. 23971497 10.3109/00498254.2013.831143

[6] Hinderling PH. Red blood cells: a neglected compartment in pharmacokinetics and pharmacodynamics. Pharmacol Rev. 1997;49(3):279–95. 9311024

[7] Biagotti S, Perla E, Magnani M. Drug transport by red blood cells. Front Physiol. 2023;14:1308632. 10.3389/fphys.2023.1308632. 38148901 10.3389/fphys.2023.1308632 PMC10750411

[8] Soichot M, Mégarbane B, Houzé P, Chevillard L, Fonsart J, Baud FJ, et al. Development, validation and clinical application of a LC-MS/MS method for the simultaneous quantification of hydroxychloroquine and its active metabolites in human whole blood. J Pharm Biomed Anal. 2014;100:131–7. 10.1016/j.jpba.2014.07.009. 25165008 10.1016/j.jpba.2014.07.009

[9] Chen F, Yang X, Li H, Zeng X, Deng Z, Wang H, et al. Improved LC–MS/MS method for the simultaneous quantification of tacrolimus and cyclosporine A in human blood and application to therapeutic drug monitoring. Biomed Chromatogr. 2023;37(12):e5751. 10.1002/bmc.5751. 37772369 10.1002/bmc.5751

[10] Roelofsen-de Beer RJAC, van Zelst BD, Wardle R, Kooij PG, de Rijke YB. Simultaneous measurement of whole blood vitamin B1 and vitamin B6 using LC-ESI-MS/MS. J Chromatogr B Analyt Technol Biomed Life Sci. 2017;1063:67–73. 10.1016/j.jchromb.2017.08.011. 28846867 10.1016/j.jchromb.2017.08.011

[11] Isberner N, Gesierich A, Balakirouchenane D, Schilling B, Aghai-Trommeschlaeger F, Zimmermann S, Kurlbaum M, Puszkiel A, Blanchet B, Klinker H, Scherf-Clavel O. Monitoring of Dabrafenib and Trametinib in serum and self-sampled capillary blood in patients with BRAFV600-mutant melanoma. Cancers. 2022;14(19):4566. 10.3390/cancers14194566. 36230489 10.3390/cancers14194566 PMC9558510

[12] Zimmermann S, Aghai F, Schilling B, Kraus S, Grigoleit GU, Kalogirou C, et al. Volumetric absorptive microsampling (VAMS) for the quantification of ten kinase inhibitors and determination of their in vitro VAMS-to-plasma ratio. J Pharm Biomed Anal. 2022;211:114623. 10.1016/j.jpba.2022.114623. 35121279 10.1016/j.jpba.2022.114623

[13] Zimmermann S, Aghai-Trommeschlaeger F, Kraus S, Grigoleit GU, Gesierich A, Schilling B, et al. Clinical validation and assessment of feasibility of volumetric absorptive microsampling (VAMS) for monitoring of nilotinib, cabozantinib, dabrafenib, trametinib, and ruxolitinib. J Pharm Biomed Anal. 2023;228:115311. 10.1016/j.jpba.2023.115311. 36841066 10.1016/j.jpba.2023.115311

[14] Ouellet D, Kassir N, Chiu J, Mouksassi M-S, Leonowens C, Cox D, et al. Population pharmacokinetics and exposure-response of trametinib, a MEK inhibitor, in patients with BRAF V600 mutation-positive melanoma. Cancer Chemother Pharmacol. 2016;77:807–17. 10.1007/s00280-016-2993-y. 26940938 10.1007/s00280-016-2993-y

[15] Raynal M, Alvarez J-C, Saiag P, Beauchet A, Brentano-Funck C, Brentano-Funck E. Monitoring of plasma concentrations of Dabrafenib and Trametinib in advanced BRAFV600^mut^ melanoma patients. Ann Dermatol Venereol. 2022;149:32–8. 10.1016/j.annder.2021.04.005. 34183171 10.1016/j.annder.2021.04.005

[16] Van der Kleij MBA, Guchelaar NAD, Meertens M, Westerdijk K, Giraud EL, Bleckman RF, et al. Reasons for non-feasibility of therapeutic drug monitoring of oral targeted therapies in oncology – an analysis of the closed cohorts of a multicentre prospective study. Br J Cancer. 2024;131(5):843–51. 10.1038/s41416-024-02789-2. 38971952 10.1038/s41416-024-02789-2 PMC11369282

[17] Prenen H, Guetens G, De Boeck G, Highley M, van Oosterom AT, de Bruijn EA. Everolimus alters Imatinib blood partition in favour of the erythrocyte. J Pharm Pharmacol. 2006;58:1063–6. 10.1211/jpp.58.8.0006. 16872552 10.1211/jpp.58.8.0006

[18] Airiau K, Turcq B, Bouchet S, Laharanne E, Vial J-P, Etienne G, et al. Dasatinib-loaded erythrocytes trigger apoptosis in untreated chronic myelogenous leukemic cells: a cellular reservoir participating in dasatinib efficiency. HemaSphere. 2018;2:3e41. 10.1097/HS9.0000000000000041. 10.1097/HS9.0000000000000041 PMC6745996 31723769

[19] Meertens M, Kerssemakers N, de Vries N, Rosing H, Steeghs N, Beijnen JH, et al. Clinical application of volumetric absorptive microsampling for therapeutic drug monitoring of oral targeted anticancer drugs. Ther Drug Monit. 2025;47:625–34. 10.1097/FTD.0000000000001315. 39996568 10.1097/FTD.0000000000001315 PMC12422603

[20] Committee for Medicinal Products for Human Use, European Medicines Agency. Assessment report. Iclusig. International non-proprietary name: PONATINIB. Procedure No EMEA/H/C/002695/0000. EMA/CHMP/220290/2013. https://www.ema.europa.eu/en/documents/assessment-report/iclusig-epar-public-assessment-report_en.pdf.

[21] European Medicines Agency. Annex 1: Summary of product characteristics. Tasigna 50 mg hard capsules, Tasigna 150 mg hard capsules, Tasigna 200 mg hard capsules. https://www.ema.europa.eu/en/documents/product-information/tasigna-epar-product-information_en.pdf.

[22] Center for Drug Evaluation and Research, Food and Drug Administration. Medical review(s). Application number: 205552Orig2s000. Reference ID: 3452364. https://www.accessdata.fda.gov/drugsatfda_docs/nda/2014/205552Orig2s000MedR.pdf.

[23] Romanski M, Zacharzewska A, Tezyk A, Glówka FK. In vivo red blood cells/plasma partition coefficient of Treosulfan and its active monoepoxide in rats. Eur J Drug Metab Pharmacokinet. 2018;43(5):565–71. 10.1007/s13318-018-0469-7. 29542019 10.1007/s13318-018-0469-7 PMC6133075

[24] R Core Team, R Foundation for Statistical Computing. R: a language and environment for statistical computing. http://www.R-project.org.

