# Supplementary material for: The extensive erythrocyte-plasma partitioning of trametinib – implications for pharmacokinetic studies and therapeutic drug monitoring
Source: Pharmacol Rep. 2026 Feb 19;78(3):920–8. doi: 10.1007/s43440-026-00840-y (PMC13275622; doi:10.1007/s43440-026-00840-y)
Supplement: Supplementary file 1 — Supplementary Material 1 [file 43440_2026_840_MOESM1_ESM.docx]

**The extensive erythrocyte-plasma partitioning of trametinib – implications for pharmacokinetic studies and therapeutic drug monitoring**

Bence János Chriszt ^1^, Zoltán Köllő ^1^, Orsolya Geda ^1^, Éva Csöndör ^1^, Róbert Farkas ^1^,
Barna Vásárhelyi ^1^, Miklós Garami ^2^, and Gellért Balázs Karvaly ^1^

^1^ Department of Laboratory Medicine, Semmelweis University

4 Nagyvárad tér, Budapest, H-1089 Hungary

^2^ Pediatric Center, Semmelweis University

7-9 Tűzoltó utca, H-1094 Budapest, Hungary

**Corresponding author:**

Gellért Balázs Karvaly

Department of Laboratory Medicine, Semmelweis University

4 Nagyvárad tér, Budapest, H-1089 Hungary

karvaly.gellert.balazs@semmelweis.hu

**Supplementary information**

**Supplementary information 1: Materials and analytical methodology**

*Preparation of solutions and calibrators.* Stock solutions containing 1 mg/mL TRT were prepared in DMSO. On each occasion, separate methanolic solutions were prepared for spiking calibrators and experimental samples. The internal standard solution (IS) contained ^13^C_6_-TRT at 50 ng/mL in acetonitrile. Plasma calibrators were prepared by spiking TRT into Chromsystems controls to achieve concentrations of 7, 14, 28, 140, 280, and 560 ng/mL. Calibrators were stored in aliquots of 75 µL at ─70°C for a maximum of 2 months.

*Preparation of samples for analysis.* Fifty µL sample (plasma, whole blood, or haemolysed blood cell pellet) was pipetted into a slot of the deproteinization plate. Two hundred µL IS was added. The plate was shaken on an Allsheng 96-well plate shaker (Lab-Ex Kft., Budapest, Hungary) at 1,100 rpm for 10 min, followed by the transfer of the supernatant into a 96-well deep-well collection plate using nitrogen 5.0 and a Presston 1000 positive-pressure manifold (Gen-Lab Kft.). The filtrate was diluted twofold with water and was submitted for analysis.

*Analysis of trametinib.* A Shimadzu Nexera X2 UHPLC – LCMS-8060 triple quadrupole mass spectrometer was used (Simkon Kft., Budapest, Hungary). Chromatographic separation was achieved using water (A) and methanol (B) as mobile phases, both containing 0.3% formic acid. The following gradient was used (%B): 0 min, 30%; 0.50 min, 30%; 3.00 min 50%; 3.01 min, 90%; 5.00 min, 90%; and 5.01 min, 30%. The mobile phase flow rate was 0.25 mL/min, and the run time was 7.00 min. The stationary phase was kept at 40°C. The sample injection volume was 1 µL. The mass spectrometer was equipped with an electrospray ion source and operated in positive mode. The interface, desolvation line, and heat block temperatures were 350 °C, 250 °C, and 400 °C, respectively. The following gas flow rates were applied: nebulizing gas, 3 L/min; heating gas, 12 L/min; and drying gas, 6 L/min. Quantitation of trametinib (m/z=615.7/490.9) and ^13^C_6_-trametinib (m/z=621.7/496.9) was effected using multiple reaction monitoring with the following settings: Q1 bias, -20.0; collision energy, -26.0; Q3 bias, -20.0; dwell time, 50 msec. System control and data evaluation were managed by the Shimadzu LabSolutions CE 1.2 software (Simkon Kft).

*Data evaluation.* The six-point calibration model was linear with 1/concentration^2^ weights between 7-560 ng/mL, obtained by plotting TRT/^13^C_6_-TRT peak area ratios against nominal TRT concentrations. The system control-data acquisition software calculated assayed TRT concentrations. Further evaluation and visualization were conducted using Microsoft Excel 365.

**Supplementary information 2: Description of the *in vitro* experiments**

*Loss of trametinib from plasma after spiking whole blood or plasma directly.* Five of the authors (B.C., E.Cs., Z.K., R.F., and G.B.K.) donated blood for the experiments. A total of 6 mL of blood was collected from each donor into two Greiner Bio-One 3-mL lavender-top collection tubes containing EDTA as anticoagulant (Greiner Bio-One Hungary Kft., Mosonmagyaróvár, Hungary) in a standard phlebotomy process by a certified phlebotomist. One tube was centrifuged at 1,100 x g for 7 min. One milliliter of plasma was transferred to a microcentrifuge tube and spiked with TRT at 25.2 ng/mL. One milliliter of whole blood collected in the other tube was spiked at the same concentration. The samples were subsequently allowed to sit at room temperature or 2-8 °C for six hours. Aliquots were taken at 0, 1, 2, 3, and 6 hours. Whole blood was centrifuged at 10,000 x g for 10 min, and the supernatant was transferred to another microcentrifuge tube for storage at ─70 °C until analysis.

*Penetration of trametinib into erythrocytes at various concentrations and temperatures.* Five of the authors (B.C., E.Cs., Z.K., R.F., and G.B.K.) donated blood for the experiments. For each experiment, a total of 3 mL of blood was collected from each donor into a single Greiner Bio-One 3-mL lavender-top collection tube containing EDTA as an anticoagulant (Greiner Bio-One Hungary Kft.) in a standard phlebotomy process by a certified phlebotomist. After gently rotating the tubes, TRT was spiked at concentrations of 20.1, 60.3, or 101 ng/mL into 2000 µL whole blood previously pipetted into a 5-mL microcentrifuge tube. A 1000-µL aliquot was cooled immediately to 2-8 °C, while the remaining blood was allowed to sit on the bench at room temperature. At 0, 20, 40, 60, and 120 min, aliquots (150 µL) were centrifuged (1,100 x g, 7 min), and the entire supernatant was transferred to a 1.5-mL microcentrifuge tube and stored at -70 °C until analysis. At 120 min, a 50-µL pellet was diluted with 100 µL water. The mixture was allowed to sit for 1 hour, was vortexed vigorously for 1 min, and stored at -70 °C until analysis.

*Relative recovery of trametinib in whole blood, plasma separated from spiked whole blood, and plasma spiked directly.* Three of the authors (E.Cs., G.B.K., and Z.K.) donated blood for the experiment. A total of 9 mL of blood was collected from each donor into four Greiner Bio-One 3-mL lavender-top collection tubes containing EDTA as an anticoagulant (Greiner Bio-One Hungary Kft.) in a standard phlebotomy process by a certified phlebotomist. After gently rotating the tubes, one tube was centrifuged at 1,100 x g for 7 min. Three 485-µL plasma aliquots were transferred to microcentrifuge tubes and were spiked with TRT at 20.1, 60.3, or 101 ng/mL. A 1.94-mL aliquot of each of the remaining three tubes of blood was also spiked at these concentrations. Immediately after spiking TRT, the first aliquots (75 µL plasma spiked directly, plasma separated from 200 µL whole blood, and 200 µL whole blood) were deep-frozen (0-minute sample). Further aliquots with these volumes were deep-frozen after 30, 60, and 120 minutes of storage at room temperature. On three further occasions, whole blood was thawed and refrozen to destroy cellular components before analysis.

*Relationship of erythrocyte-plasma partitioning with trametinib concentration and blood temperature.* Two authors (E.Cs. and G.B.K.) donated blood for the experiment. A total of 9 mL of blood was collected from each donor into four Greiner Bio-One 3-mL lavender-top collection tubes containing EDTA as an anticoagulant (Greiner Bio-One Hungary Kft.) in a standard phlebotomy process by a certified phlebotomist. After gently rotating the tubes, one tube was centrifuged at 1,100 x g for 7 min. Three 485-µL plasma aliquots were transferred to microcentrifuge tubes and were spiked with TRT at 20.3 ng/mL (sample donated by E.Cs.) or 62.2 ng/mL (sample donated by G.B.K.). TRT was also spiked at concentrations of 26.4 ng/mL (samples donated by E.Cs.) or 79.3 ng/mL (samples donated by G.B.K.) into 1500 µL whole blood previously pipetted from each of the remaining three tubes into a 5-mL microcentrifuge tube. An aliquot of whole blood (150 µL) was centrifuged at 10,000 x g for 10 min, and the supernatant was transferred to another microcentrifuge tube for storage at ─70 °C until analysis. An aliquot of plasma (65 µL ) was also deep-frozen.

A biphasic experiment was subsequently conducted with the spiked whole blood and plasma samples. In the first phase, 1.2 mL spiked whole blood and 0.5 mL spiked plasma were shaken at 38 °C (300 rpm, condition A), or allowed to sit at RT (condition B) or 2–8 °C (condition C) for 2 hours. In the second phase, samples previously kept at 38 °C were allowed to sit at RT (condition A🡪condition B), those previously kept at RT were cooled to 2-8 °C (condition B🡪condition C), and refrigerated samples were warmed to 38 °C (condition C🡪condition A) for another 2 hours. Plasma aliquots (65 µL) were separated from 150 µL whole blood at 0.5, 1.0, 2.0, 2.5, 3.0, and 4.0 hours after centrifugation of the blood samples at 10,000 x g for 10 min, and were kept at -70 °C until analysis. In addition, plasma-spiked samples (65 µL each) were transferred to microcentrifuge tubes subsequently deep-frozen.

**Supplementary information 3: Analytical method validation in human plasma**

*Assay selectivity and specificity.* TRT and ^13^C_6_-TRT peaks were evaluated in 15 independent human plasma samples, allocated for disposal following diagnostic testing, and prepared for analysis using deproteinization approaches. Analytical selectivity was investigated using 50 µL plasma and 200 µL acetonitrile. Specificity for the TRT and ^13^C_6_-TRT was evaluated by adding 200 µL IS to 50 µL plasma, or 200 µL acetonitrile to 50 µL plasma spiked with TRT at 550 ng/mL. Selectivity and specificity were judged acceptable for TRT if the peak areas observed in samples not containing TRT did not exceed 20% of that obtained in the low-end calibrator (7 ng/mL) run along. The results were accepted for ^13^C_6_-TRT if the peak areas in samples without the internal standard did not exceed 5% of the peak area in the low-end calibrator.

*Sample carryover.* Alternating injections of the high-end calibrator (550 ng/mL) and a serum blank were performed over three injection cycles. The experiment was considered acceptable if the TRT and ^13^C_6_-TRT peak areas in the blanks did not exceed 20% and 5% of the mean peak areas observed in the ion chromatograms of the prepared high-end calibrators, respectively.

*Calibration model.* The calibration was based on the correlation between the analyte/internal standard peak area ratios and the concentration. Six calibration levels were prepared (7, 14, 28, 140, 280, and 560 ng/mL). Calibration models obtained in each run by plotting TRT/^13^C_6_-TRT peak area ratios against concentrations were required to be linear, with the determination coefficient (r^2^) exceeding 0.9900 for a weighted linear regression with 1/concentration^2^ weights. Back-calculated accuracies of TRT concentrations determined in the calibrators were required to be 85-115%, except for the low-end calibrator (80-120%). Based on these, calibration based on at least 5 points meeting the requirements was accepted.

*Matrix effect.* Eight independent human plasma samples, allocated for disposal after diagnostic testing, were spiked with TRT at 15.3 or 454 ng/mL. In addition, three samples showing visual signs of lipaemia, icterus, or haemolysis were prepared after being spiked with TRT at 20.1 or 504 ng/mL. Each matrix was prepared in three parallel samples by adding 200 µL IS to 50 µL plasma for deproteinization.

*Accuracy and precision.* Fifteen independent human plasma samples, allocated for disposal after diagnostic testing, were spiked with TRT at 7.29, 18.2, 233, or 474 ng/mL. The accuracy and precision of the TRT assay were determined in all samples on the day of preparation, and in five randomly selected matrices on the subsequent two days. At 18.2, 233, or 474 ng/mL spiking levels, the ranges of acceptability of accuracy and precision were 85-115% in terms of analyte recovery and 0–15% in terms of relative standard deviation, respectively. For samples spiked at the lowest level, the acceptibility ranges were 80-120% and 0-20%, respectively.

**Supplementary information 4: Results of method validation in plasma**

*Assay selectivity and specificity; sample carryover.* When plasma samples were deproteinized by adding acetonitrile, no TRT peak was detected in blanks, and no ^13^C_6_-TRT peak was observed when samples were spiked with TRT. Similarly, no TRT peaks were observed in blank samples prepared by adding IS for deproteinization. No sample carryover was observed.

*Calibration model*. A linear calibration model with 1/concentration^2^ weights proved most suitable for the quantitation of TRT. The calibration equations and the performance characteristics obtained during method validation are shown in Supplementary table S1. R^2^, determination coefficient. The slope was 0.00060±0.00006 (relative standard deviation: 10.8%). The confidence interval for the intercept was –0.000409-0.000013 (p=0.080 for the difference from 0).

**Supplementary table S1. Characteristics of the calibration models used for TRT method validation.**

| **Validation experiment no.** | **Intercept** | **Slope** | **R^2^** | **Back-calculated accuracy** |
| --- | --- | --- | --- | --- |
| 1 | -0.000355178 | 0.0005266621 | 0.9994 | 97.5-103% |
| 2 | -0.000441339 | 0.000559351 | 0.9992 | 97.8-104% |
| 3 | -0.000128987 | 0.000561857 | 0.9992 | 96.8-103% |
| 4 | -0.000059454 | 0.000696752 | 0.9985 | 95.3-104% |
| 5 | -0.000006208 | 0.000655491 | 0.9990 | 96.7-103% |

*Matrix effects.* The mean absolute TRT recoveries were 88.1-95.3% and 92.4-100.6% at the low and high spiking level, respectively (Supplementary table S2). In lipaemic, icteric and hemolytic samples, the mean absolute recoveries were 104.5-107.5% and 106.3-113.7%, 103.7-111.2% and 111.1-113.5%, as well as 102.5-116.8% and 110.4-111.4%, respectively (Supplementary table S3).

**Supplementary table S2. Investigation of the matrix effect by using eight independent human plasma matrices. TRT was spiked at 15.3 or 454 ng/mL. Each matrix was processed in three parallels.**

| **Matrix** | **Low spiking level (15.3 ng/mL)** | | **High spiking level (454 ng/mL)** | |
| --- | --- | --- | --- | --- |
|  | **Mean concentration (±Standard deviation, ng/mL)** | **Mean absolute recovery (±relative standard deviation, %)** | **Mean concentration (±Standard deviation, ng/mL)** | **Mean absolute recovery (±relative standard deviation, %)** |
| **A** | 14.2±0.29 | 92.5±2.0% | 428±23.4 | 94.2±5.5% |
| **B** | 13.5±0.65 | 88.1±4.8% | 446±4.45 | 98.4±1.0% |
| **C** | 13.9±0.20 | 90.8±1.4% | 452±9.86 | 99.7±2.2% |
| **D** | 14.6±0.17 | 95.1±1.2% | 441±5.79 | 97.1±1.3% |
| **E** | 13.8±0.23 | 90.1±1.7% | 454±5.75 | 100.0±1.3% |
| **F** | 14.6±0.69 | 95.3±4.7% | 419±16.7 | 92.4±4.0% |
| **G** | 14.2±0.72 | 92.5±5.1% | 456±11.1 | 100.6±2.4% |
| **H** | 14.5±0.67 | 94.7±4.6% | 443±5.30 | 97.6%±1.2% |

**Supplementary table S3. Investigation of the matrix effect in lipaemic, icteric and haemolytic plasma samples. The spiking levels were 20.1 and 504 ng/mL: Each matrix was processed in three parallels.**

| **TRT concentration** | | | | | | | | |
| --- | --- | --- | --- | --- | --- | --- | --- | --- |
| **Matrix identifier** | **20.1 ng/ml** | **504 ng/ml** | **Matrix identifier** | **20.1 ng/ml** | **504 ng/ml** | **Matrix identifier** | **20.1 ng/ml** | **504 ng/ml** |
| **Lipaemic samples** | | | **Icteric samples** | | | **Samples with haemolysis** | | |
| **Matrix LipA** | 107.5±5.00% | 106.3±2.3% | **Matrix IctA** | 111.2±3.5% | 113.5±0.8% | **Matrix HaeA** | 102.5±0.2% | 111.4±5.1% |
| **Matrix LipB** | 104.5±16.4% | 113.7±3.3% | **Matrix IctB** | 109.6±8.6% | 111.1±1.1% | **Matrix HaeB** | 109.5±5.7% | 110.4±1.4% |
| **Matrix LipC** | 107.0±13.0% | 110.8±2.9% | **Matrix IctC** | 103.7±2.2% | 112.6±2.8% | **Matrix HaeC** | 116.8±4.6% | 110.8±1.9% |

*Accuracy and precision.* The within-day accuracy was 98.6-103%, while the between-day accuracy was 108-112% (Supplementary table S4). No outlier results were detected.

**Supplementary table S4. Results of the within-run and between run accuracy and precision experiment.**

| **Experiment** | **TRT concentration spiked to plasma** | | | |
| --- | --- | --- | --- | --- |
|  | **7.29 ng/mL** | **18.2 ng/mL** | **233 ng/mL** | **467 ng/mL** |
|  | **Mean absolute recovery±relative standard deviation (%)** | | | |
| Within-run (n=15) | 103±8.0 | 101±5.2 | 101±3.1 | 98.6±2.2 |
| Between run (n=25) | 112±10.2 | 108±7.4 | 108±6.4 | 108±7.5 |
